# *FRIZZLE PANICLE* (*FZP*) regulates rice spikelets development through modulating cytokinin metabolism

**DOI:** 10.1186/s12870-023-04671-4

**Published:** 2023-12-16

**Authors:** Wei Wang, Wenqiang Chen, Junmin Wang

**Affiliations:** 1 Zhejiang Academy of Agricultural Sciences, Hangzhou, 310021 China; 2 Engineering Research Center of Ecology and Agricultural Use of Wetland, Ministry of Education/College of Agriculture, Yangtze University, Jingzhou, 434025 Hubei China

**Keywords:** Rice, Grain number, FZP, DST-OsCKX2 module, Cytokinin

## Abstract

**Background:**

The number of grains per panicle is an important factor in determining rice yield. The DST-OsCKX2 module has been demonstrated to regulate panicle development in rice by controlling cytokinin content. However, to date, how the function of DST-OsCKX2 module is regulated during panicle development remains obscure.

**Result:**

In this study, the *ABNORMAL PANICLE 1* (*ABP1*), a severely allele of *FRIZZY PANICLE* (*FZP*), exhibits abnormal spikelets morphology. We show that FZP can repress the expression of *DST* via directly binding to its promotor. Consistently, the expression level of *OsCKX2* increased and the cytokinin content decreased in the *fzp* mutant, suggesting that the *FZP* acts upstream of the DST-OsCKX2 to maintain cytokinin homeostasis in the inflorescence meristem.

**Conclusions:**

Our results indicate that *FZP* plays an important role in regulating spikelet development and grain number through mediating cytokinin metabolism.

**Supplementary Information:**

The online version contains supplementary material available at 10.1186/s12870-023-04671-4.

## Background

Rice (*Oryza sativa*) is one of the most important cereal crops in the world, providing the main energy for more than half of the world's population [1, 2]. High and stable rice yield is very important for food security, and has always been the focus of rice breeding and improvement. The composition of rice yield consists of three factors, tiller number, the filled-grains number per panicle, and grain weight [3]. Among them, number of grains per panicle is a relatively flexible component with the largest contribution to yield [4]. Rice reproductive development starts from inflorescence meristem norms, followed by branch meristem, spikelet meristem (SM) and floral meristem (FM) [5, 6]. The inflorescence meristems that form spikelets, flowers, and glumes are important to determination of grain number and size [7, 8]. Currently, several genes have been shown to determine panicle branching and spikelets number by controlling the size and activity of the meristem [9–18]. The activity of reproductive meristems plays an important role in determining rice yield [19].

Cytokinin is a specific hormone that affects plant growth and development, and plays an important role in regulating shoot apical meristem (SAM) in plants [9, 20–22]. Reducing the level of endogenous cytokinin or inhibiting its signal transduction will reduce the activity of SAM, and conversely will increase the activity of SAM [23–28]. Cytokinin oxidase/dehydrogenase (CKX), which can irreversibly degrade cytokinins, is involved in mediating the balance of endogenous cytokinins in plants [29]. The CKXs were encoded by 11 gene in rice [30]. The major QTL locus that negatively regulates grain numbers, *Grain number 1a* (*Gn1a*), encodes cytokinin oxidase/dehydrogenase 2 (OsCKX2), which can catalyze the degradation of active cytokinins [11]. Decreased expression of OsCKX2 in transgenic rice harboring antisense OsCKX2 cDNA resulted in increased grain number in the panicle [11]. Increasing cytokinin level in florescence meristems by reducing or lost function of *OsCKX2* expression has been successfully used in rice breeding practice to increase seed yield [11]. Transcription factor DROUGHT AND SALT TOLERANCE (DST) regulates the activity of florescence meristems by regulating the expression of *CKX2* through directly binding to its promoter [14]. ERECTA1 (OsER1) activates the OsMKKK10-OsMKK4-OsMPK6 cascade, and the activated OsMKKK10-OsMKK4-OsMPK6 directly phosphorylates DST, thereby increasing the expression level of OsCKX2, indicating that OsER1 regulates the development of rice spikelet by mediating cytokinin metabolism [4]. A recent study showed that Mediator subunit 25 (MED25) acts as an interacting coactivator of DST, in the same pathway as the DST-OsCKX2 module to regulate spikelet number per panicle [18]. However, the exact molecular mechanism of how the DST-CKX2 module regulates inflorescence development through cytokinin metabolism is still not clear.

The spikelet identity gene, *FRIZZY PANICLE* (*FZP*), is involved in the transition from spikelet to floral meristem identity, which contains an APETALA2/ETHYLENE RESPONSE FACTOR (AP2/ERF) domain transcription factor. FZP is an ortholog of the maize transcription factor BD1 [10, 31, 32]. Recent studies have revealed that OsBZR1 and OsARFs can regulate the expression of *FZP* at the transcriptional stage [33]. Deletion of 4-bp tandem repeats at about 2.7 kb upstream of FZP affected its binding activity to auxin reaction factor, resulting in decreased FZP expression level and increased the number and yield of secondary branches [34]. In another study, OsPTB1/2 can mediate *FZP* translational repression by interacting with CUREs in the 3’ UTR of *FZP* mRNA, leading to changes in the number of secondary branches and the grain number per panicle [35]. However, the precise molecular mechanism of FZP regulating grain number needs to be further improved.

In this study, we identified an *ABNORMAL PANICLE 1* (*ABP1*) gene, which regulates spikelets development and grain yield in rice. Map-based cloning revealed that *ABP1* was identical to *FRIZZY PANICLE* (*FZP*). Molecular evidence shows that FZP directly binds to the motif AGCCGCC in the *DST* promoter that represses its expression. Consequently, the expression level of *OsCKX2* was upregulated and the level of cytokinin decreased in the inflorescence meristem of *fzp* mutant, which reduces the SAM activity and the grain number of per panicle. Our findings reveal that *FZP* regulates grain number by negatively controlling the function of DST-OsCKX2 module, which provides novel insight into the regulation of cytokinin homeostasis in rice.

## Results

### *abp1* mutant displays abnormal spikelet morphology

**Fig. 1:**
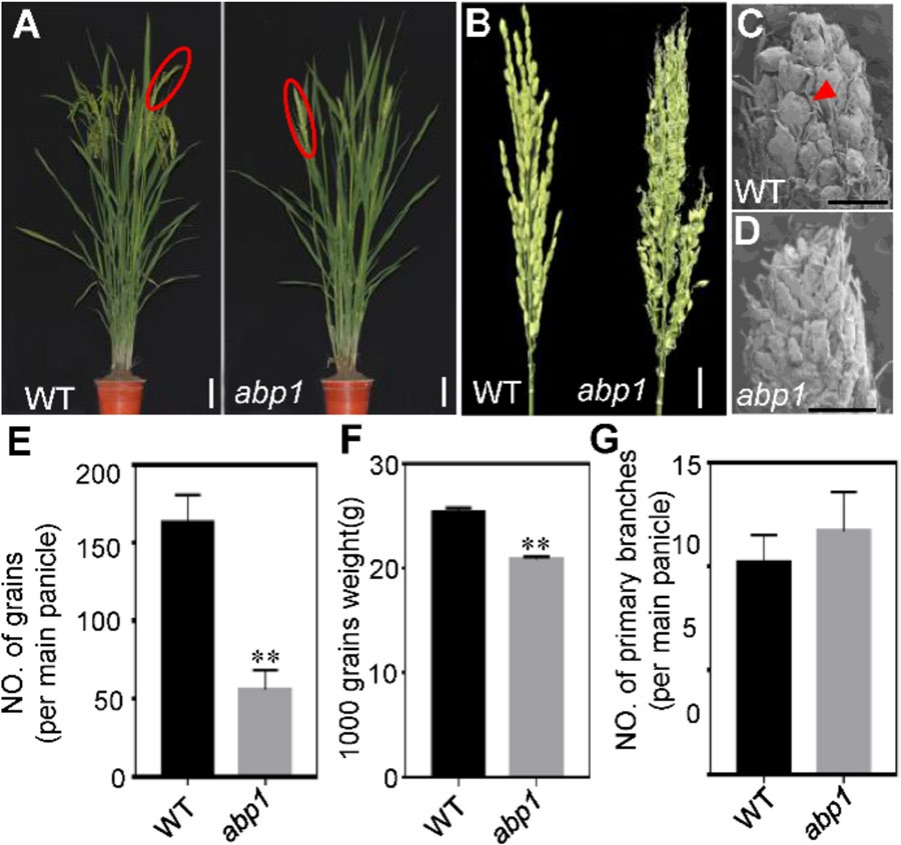
Phenotypic analysis of spikelet in wild type and *abp1* mutant. **A** Plants morphology of wild type and *abp1* after heading, bar = 10 cm. **B** Main panicle of wild type and *abp1*, bar = 2.5 cm. **C**, **D** Scanning electronic microscope analysis of wild type and *abp1* panicle at early differentiation stage, the red triangle indicates the floret meristem. bar = 500 μm. **E** Statistical analysis of grain number per main panicle. **F** Statistical analysis of 1000-grain weight. **G** Statistical analysis of primary branches number per main panicle. Data are means ± SD from three biological replicates. ** means significant difference between wild type and *abp1* mutant as determined by the Student’s *t* test (*P* < 0.01)

To better understand the mechanism of rice panicle development, we isolated an abnormal spikelets mutant named *abnormal panicle 1* (*abp1*) from an EMS-treated mutant pool of the elite *indica* rice variety YK17 (Zhongjiazao17). The *abp1* mutant was similar to the wild type in plant morphology (Fig. 1A), but the spikelets were replaced by numerous higher order branches in the panicle of *abp1* which should have grown on the primary branches and secondary branches (Fig. 1B and Supplemental Fig. S1A, B). Scanning electronic microscopy (SEM) examination also suggested that the differentiation of the floral primordia was not differentiated in *abp1* panicle but replaced by higher-order branch primordia (Fig. 1C-D and Fig. S1C-D). Eventually, grain number per main panicle of *abp1* was reduced by 66% compared to wild type, and the grains were also smaller than the normal plants (Fig. 1E-G and Fig. S2).

### *ABP1* is a new allele gene of *FZP*

**Fig. 2:**
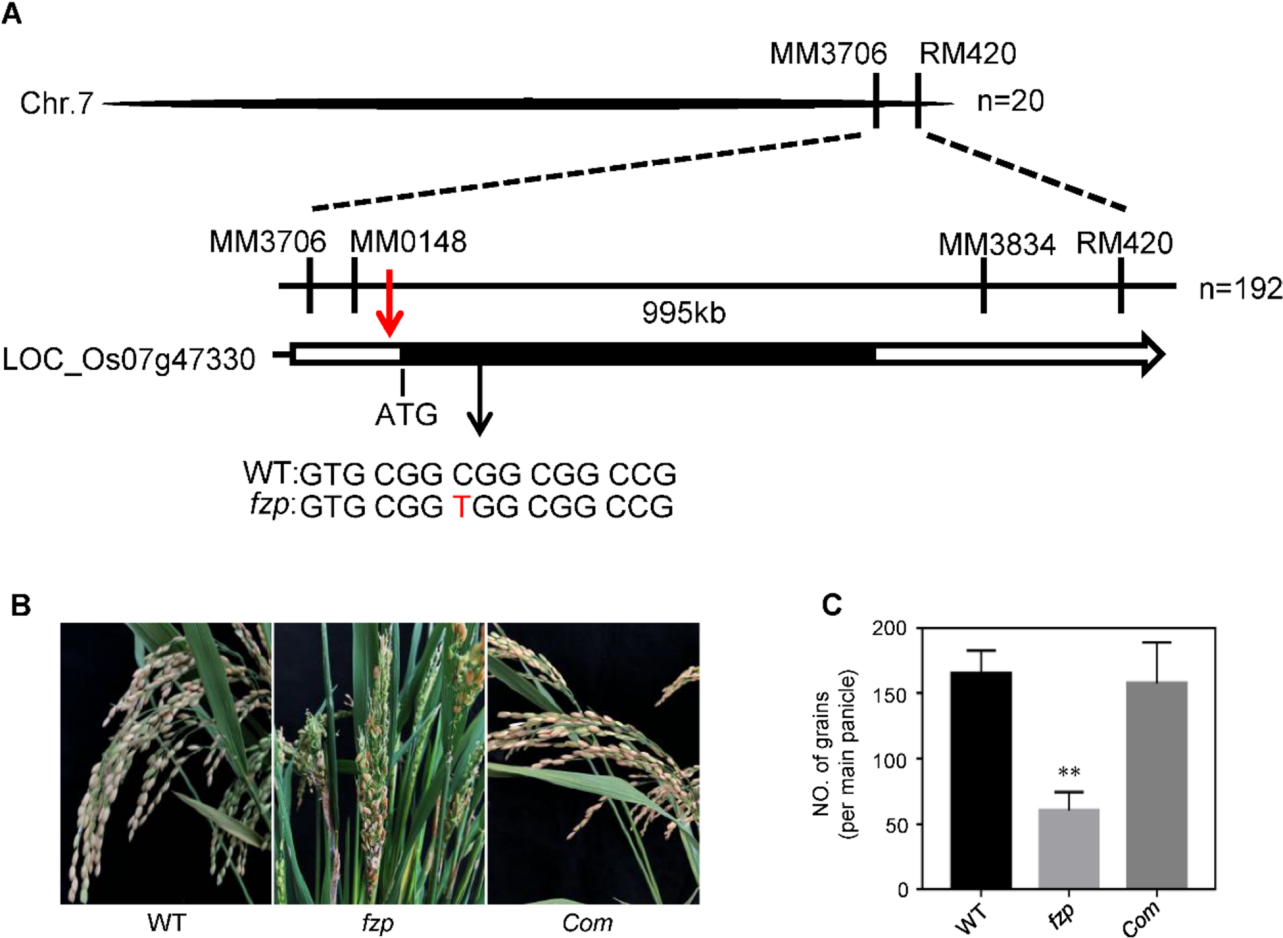
Map-based cloning of *FZP*. **A** *FZP* was mapped in a 995 kb region between markers MM0148 and MM3834 on chromosome 7. **B** Genetic complementation test of *FZP*. Panicles morphology of WT, *fzp* and the genetic complementation lines (*Com*). **C** Comparison of grain number per main panicle between WT, *fzp* and *Com*. Data are shown as means ± SD (*n* = 10)

**Fig. 3:**
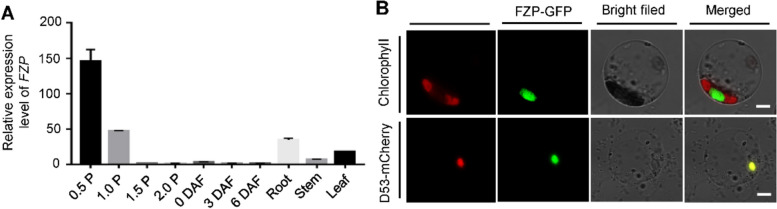
Expression pattern of *FZP*. **A** qRT-PCR analysis of expression pattern of *FZP* in different tissues, development panicles and seeds. 0.5 P, young panicles (≤ 0.5 cm); 1.0 P, young panicles (0.5–1 cm); 1.5 P, young panicles (1–1.5 cm); 2.0 P, young panicles (1.5–2 cm); DAF, Days after fertilization. Data are shown as means ± SD of three biological replicates. **B** Subcellular localization of FZP-GFP fusion protein in rice protoplasts. D53-mCherry was served as a nuclear marker, Bars = 5 μm

To determine the *ABP1* gene, we adopted map-based cloning strategy, the genetic populations of *abp1* and D50 (*O. sativa, ssp. japonica*) were constructed. The gene was mapped in the chromosome 7 primarily, and delimited to a 995 kb region between SSR markers MM0148 and MM3834, which harbors a *Frizzy panicle* (*FZP*, *LOC_Os07g47330*) gene (Fig. 2A). Genomic sequence analysis revealed that ‘C’ was replaced by ‘T’ on the only ORF of *FZP*, which led to an amino acid substitution of Arg-63 to Trp-63 in the *abp1* mutant (Fig. 2A and Fig. S3). To confirm the genetic mapping result, a DNA fragment containing the promotor and genomic was introduced to the *abp1* callus. 24 transgenic lines were obtained and all showed the wild type morphology in panicle, therefore, *FZP* is the target gene and responsible for the mutant phenotypes (Fig. 2B-C and. Fig. S4). qRT-PCR assay showed that *FZP* was expressed in all rice tissues examined, with higher level in young panicle shorter than 0.5 cm (Fig. 3A). To examine subcellular localisation of FZP, vectors expressing fused GFP and the full-length cDNA of FZP were generated and co-expressed with the nucleus marker D53- mCherry in protoplasts [36, 37]. We found that the signals of FZP-GFP co-localized with D53- mCherry in the nucleus (Fig. 3B).

### FZP directly represses the transcription of *DST*

**Fig. 4:**
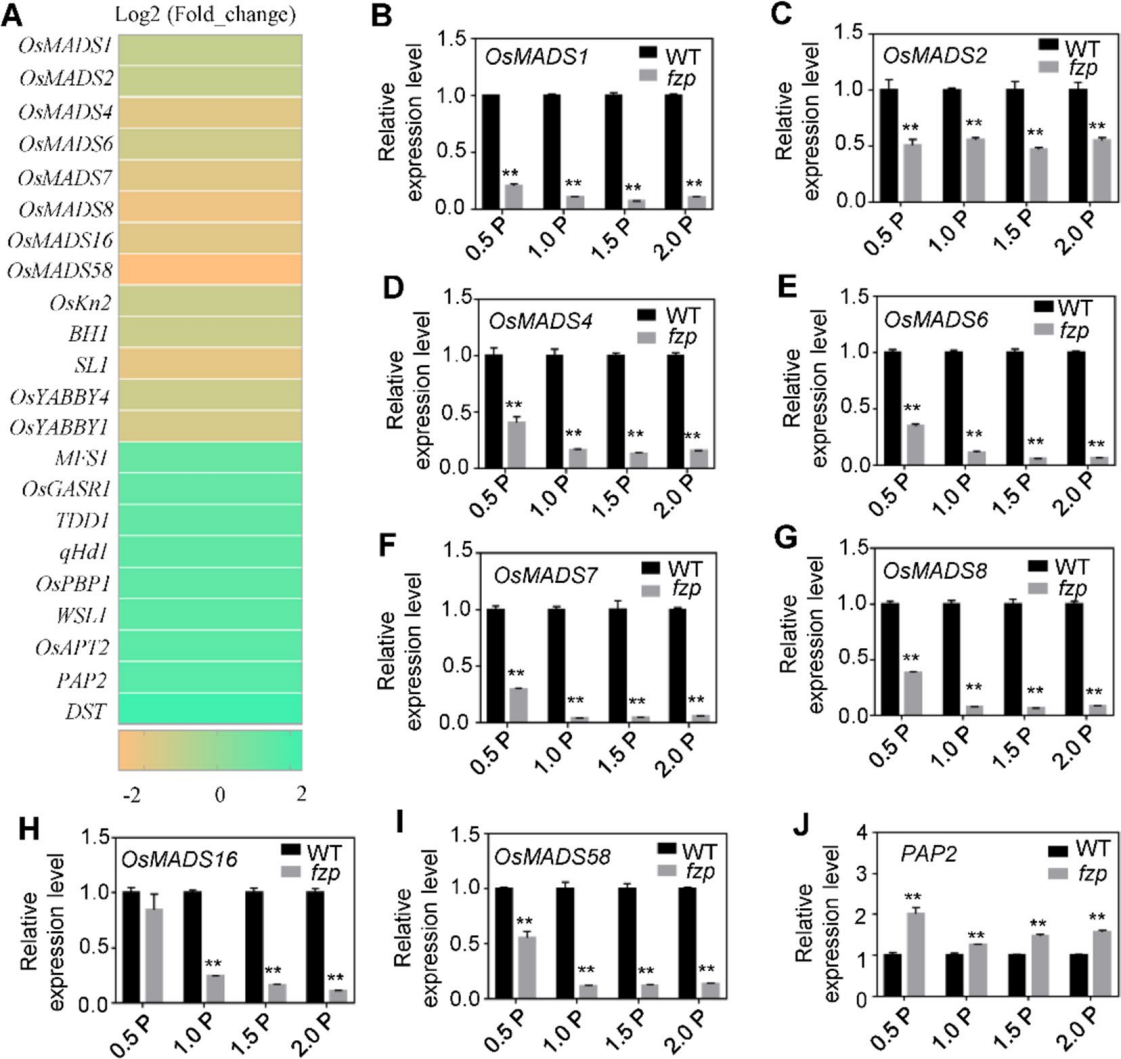
Comparison and analysis of differentially expressed of genes related to spikelets development between WT and *fzp*. **A** Expression heat map of genes related to spikelets development. **B**-**J** Transcript levels of *OsMADS-box* genes in the developing panicle of WT and *fzp* revealed by RT-PCR. Data are means ± SD for three biological replicates. The asterisk indicates the difference between WT and *fzp* determined by Student’s t-test (**P < 0.01)

Since *FZP* encodes a transcription factor containing an AP2/ERF domain, in order to improve its regulation mechanism and identify the downstream regulators, we performed RNA-sequencing (RNA-seq) experiments was in the young panicle (≤ 0.5 cm) of *fzp* and wild-type. A total 578 differentially expressed genes (DEGs) were identified, including 161 downregulated and 417 upregulated in *fzp* (Table S1). Gene Ontology (GO) analysis revealed that gene group are enriched in “response to abiotic”, “thylakoid” and “chlorophyll binding” interms of “biological process,” “cellular component,” and “molecular function,” respectively (Fig. S5A). The kyoto encyclopedia of genes and genomes (KEGG) pathways indicated that the DEGs are enriched in “metabolic pathways” and “biosynthesis of secondary metabolites” (Fig. S5B). To verify the RNA-seq results and find the downstream regulators of *FZP*, we further conducted RT-PCR analysis on 9 *OsMADS-box* DEGs in developing panicle cDNA samples, which are related to the development of inflorescence. Most *OsMADS-box* genes were significantly down regulated in mutants, especially *OsMADS7* and Os*MADS8*, which were reported to lose floral determinacy after the expression were both down regulated at the same time [38]. The results were consistent with the inclination of RNA-seq, suggesting that the RNA-seq results are highly reliable (Fig. 4B-J).

**Fig. 5:**
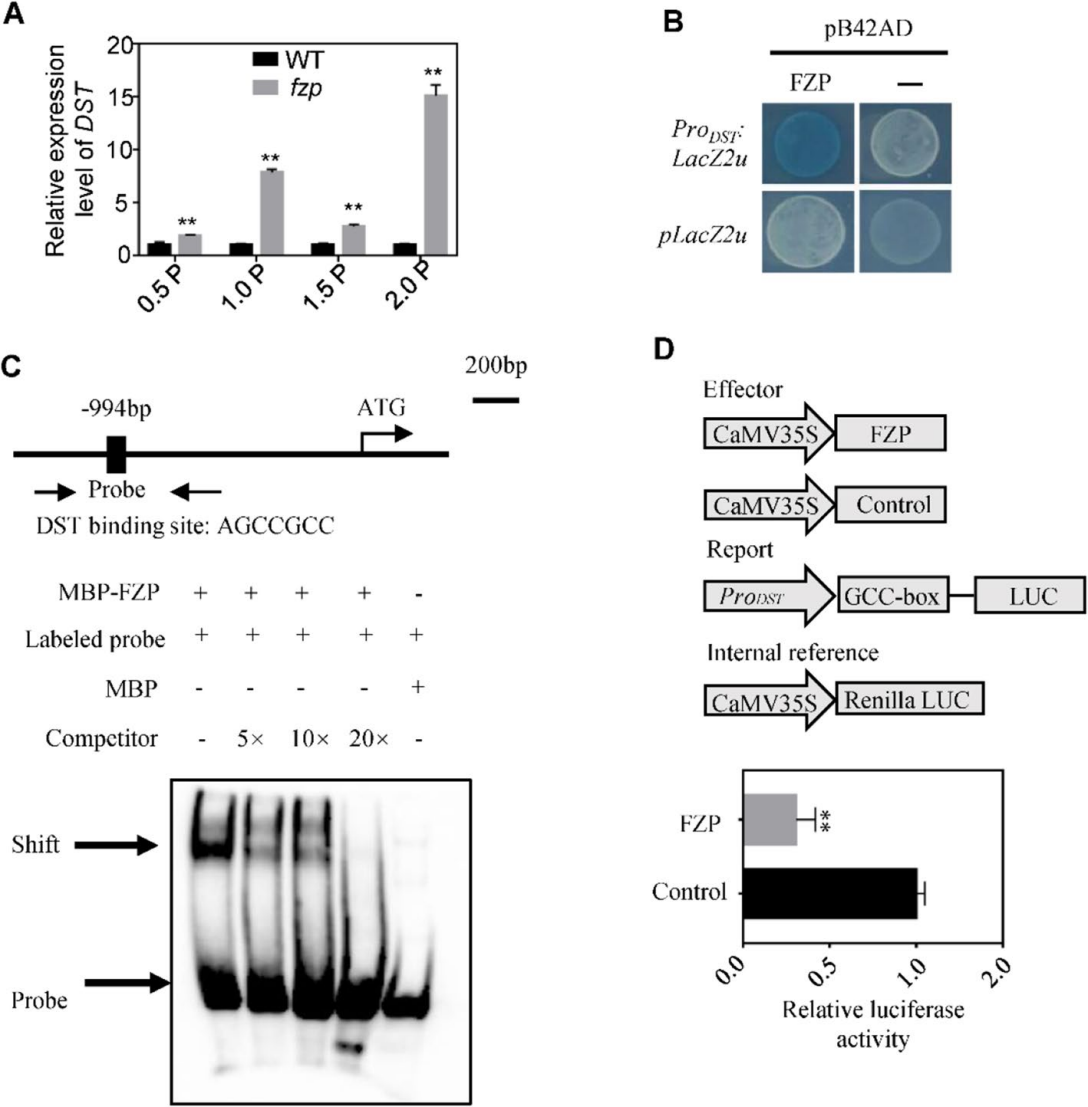
FZP binds directly to the promoter of *DST* and suppresses its expression. **A** RT-PCR analysis of *DST* expression level in development panicle of wild type and *fzp*. Values are presented as means ± SD with three biological replicates, and the statistically significant differences were determined by Student’s t-test (**P < 0.01) **B** Y1H showing that FZP could binding to the promoter of *DST*. **C** EMSA assay showing that FZP could binding to the motif AGCCGCC of *DST* promoter directly. The excess unlabeled probes were used as competitors. **D** Luciferase reporter assay showing that FZP suppresses the expression of *DST* in rice protoplasts. Data are shown as means ± SD with three biological replicates. **, *P* < 0.01 by Student’s t-test

Meanwhile, we selected the differential genes related to panicle development in DEGs for analysis, and found that the expression levels of majority tested genes were significantly upregulated in *fzp* (Fig. 4A). Particularly, the transcription of *DST* was dramatically elevated (Fig. 5A), it has been known that regulates the activity of florescence meristems by binding to the promoter of *CKX2* and inducing its expression [14]. The differential expression of *DST* in *fzp* and YK17 led us to test whether it is the downstream target of FZP, so we conducted RT-PCR analysis first, as shown in Fig. 5A, *DST* showed significant upregulation in *fzp* compared with YK17 in developing panicles. To confirm that *DST* is the downstream target gene of FZP, we performed a yeast one-hybrid (Y1H) assay, the result showed that FZP could bind to the promotor of *DST* (Fig. 5B). A previous study reported that ERF domain binds specifically to AGCCGCC motif [39]. The *DST* promoter harbors a AGCCGCC motif staring 994 bp upstream of the start codon (Fig. 5C). We purified MBP-FZP proteins for electrophoretic mobility gel shift assay (EMSA), the result showed FZP was able to bind to the GCC-box in the promoter regions of *DST*, while the shifted band signal was substantially weakened when non-labeled (Fig. 5C). To verify whether FZP could activate/repress the expression of *DST*, a dual-luciferase (LUC) assay was conducted by using *proDST: LUC* as a reporter. In rice protoplasts, effectors *35S: FZP* significantly repressed the transcription of the reporter compared with the negative control (Fig. 5D). These results indicated that *DST* is the direct downstream target gene of FZP and the expression level was repressed by FZP.

### FZP controls grain number by regulating cytokinin metabolism

**Fig. 6:**
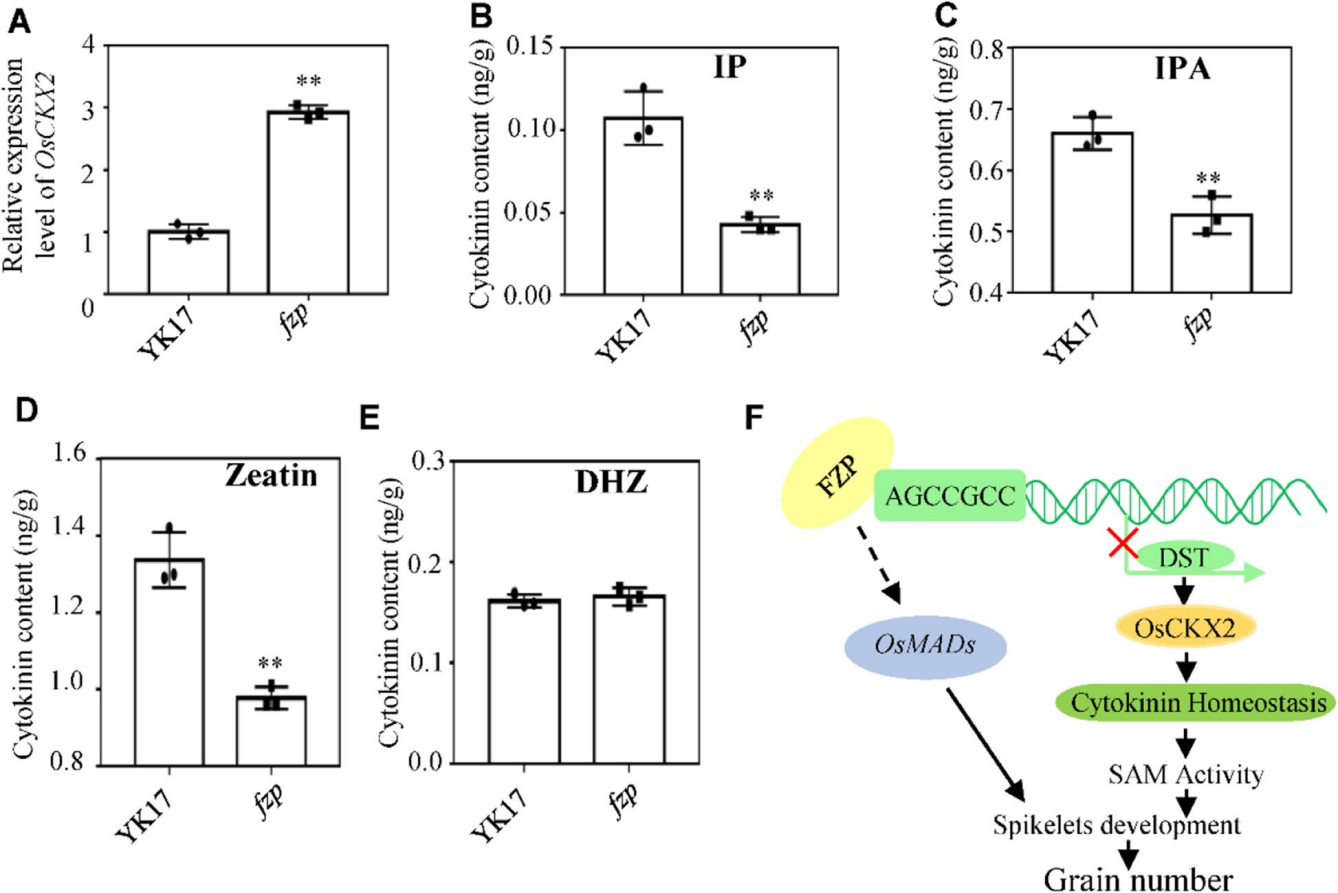
The function of FZP in Cytokinin Metabolism. **A** Transcript level of *OsCKX2* in the inflorescence meristems of WT and *fzp* revealed by qRT-PCR. **B**-**E** Comparison endogenous CK level in the inflorescence meristems of WT and *fzp*, IP, N6-(2-Isopentenyl) adenosine, IPA, isopentenyl adenosine, DHZ, DL-Dihydrozeatin, data are shown as means ± SD of three biological replicates. ** *P* < 0.01 by the Student’s *t* test. **F** Proposed model of the role of FZP in regulating the number of grains

DST have been reported to control inflorescence meristem activity by regulating the expression of *OsCKX2* [14]. The decrease of *OsCKX2* expression level resulted in the accumulation of cytokinin in the inflorescence meristem, which increased the number of reproductive organs and increased grain yield [11]. These results suggested that *FZP* may control rice spikelet development and grain number through regulating cytokinin metabolism. To test this, RT-PCR was performed first, the result showed that *OsCKX2* was upregulated in *fzp* compared with wild-type in 0.5 cm young panicles (Fig. 6A). Thus, the endogenous cytokinin levels in 0.5 cm young panicles was assayed and found that the content of IP, IPA and zeatin decreased 60%, 25% and 27% respectively, while DHZ was almost unchanged in the mutant (Fig. 6B-E), confirming that FZP is required for controlling cytokinin metabolism. Together, FZP directly represses the transcription of *DST*, the expression level of *DST* and *OsCKX2* were upregulated in the *fzp* mutant, resulting in the decrease of cytokinin content in the young panicle meristem, and the activity of SAM was affected (Fig. 6F).

## Discussion

### FZP plays a positive role in determining spikelets number

Rice grain number per panicle is the most important factor for increasing grain yield [3]. Development of panicle architecture is a complex process, which is decided by several genetic factors, the exact molecular mechanism is still not clear enough. When rice is transitioning from the vegetative to the reproductive phase, the shoot apical meristem (SAM) changes into an inflorescence meristem (IM), and then form branch primordia and subsequently generate spikelets meristems (SMs). The transformation from branch primordium to spikelets primordium determines the structure of the panicle and the grains number, many genes are involved in this process. *TAW1 (TAWAWA 1)* controls panicle development by regulating the activities of florescence meristem activity, mutation in *TAW1* shows the differentiation of spikelet meristem was delayed, the differentiation time of branch meristem was prolonged, and significantly increases the number of secondary branches and spikelets in rice [40]. Overexpression of *RCN1*and *RCN2* (*RICE CENTRORADIALIS*) delayed the transition from branch meristem to spikelets meristem, resulting in an increased number of branches and the grains number of per panicle [41]. *FZP* is involved in the differentiation of spikelets meristem and prevent the formation of axillary meristem, has become a marker gene for the development of spikelet meristem in grasses, several *FZP* alleles have been cloned, Our results showed that *ABP1* is a severely active allele of *FZP*, grain number per main panicle of *abp1* was reduced by 66% compared to wild type, the floral primordia was not differentiated in *abp1* panicle but replaced by higher-order branch primordia, and the grains were also smaller than the normal plants (Fig. 1). This severe phenotype might be caused by the single base substitution in ERF domain and the amino acid sequence was changed (Fig. 2 and Fig. S3). The regulation of *FZP* on panicle development has a certain flexibility, moderately reduced its expression can increase the number of branches while ensuring the normal development of spikelets, which has important potential for high yield breeding.

### FZP encodes an AP2/ERF domain transcription factor

The APETALA2/ethylene-responsive element binding factors (AP2/ERF), a highly conserved DNA domain, are a class of plant-specific transcription factors [39]. Previous studies have reported that the ERF transcription factors can specifically bind to *cis*-acting GCC-box, and this type TFs were essential regulators of plant growth and development and hormonal regulation, such as control of spikelet meristem fate and kernel development [42]. A few examples are *INDETERMINATE SPIKELET1* (*IDS*) in maize belonged to AP2/ERF family, specifies determinate fates by suppressing indeterminate growth within the spikelets meristem, and which is required for timely conversion of spikelets meristem to floral meristems timely [43]. Rice *MULTI-FLORET SPIKELET1* (*MFS1*) belong to AP2/ERF family, plays an important role in the regulation of spikelets meristem determinacy and floral organ identity, mutation in *MFS1* resulted in an extra hull-like organ and an elongated rachilla [44]. *FZP* also encodes an AP2/ERF-type transcription factor, ERF domain can be divided into three classes according to the amino acid characteristics, *FZP* belongs to the class II, and transient analysis shows that the class II members usually have transcriptional repressor activity [10]. However, the downstream genes of FZP have not yet been clarified. Distinct from the previous studies, this study detected that the expression level of *DST* which controlling the number of grains per panicle was significantly up-regulated in *fzp* by analysis the transcriptome data, and the promoter of *DST* also contained the *cis-acting* GCC-box (Fig. 4). We revealed that the transcription of *DST* is directly repressed by FZP with solid pieces of evidence from the in vitro and in vivo experiments (Fig. 5). Our results are consistent with the earlier reports that FZP belongs to the class II and shows transcriptional repressor activity [10]. Elucidate this regulatory pathway may help to improve the mechanism of FZP regulation on rice spikelets meristem development and grain number.

### FZP regulates grain number dependent on DST-OsCKX2 Module

Cytokinins (CTK), an important plant hormone, was first discovered in tobacco tissue culture, CTK are widely involved in regulating plant growth, development and response to the environment, such as promoting cell division, removing apical dominance and shortening the transition time from vegetative growth to reproductive growth [30]. The shoot apical meristem (SAM) is transformed into the inflorescence meristem (IM) when rice enters the reproductive growth stage, IM produces branching meristems (BMs), which can initiate secondary BMs to form lateral branches and spikelet meristems (SM), so the inflorescence traits directly affect crop yield. CTK plays an important role in regulating the activity of SAM and IM. During the development of floral meristem, increasing the concentration of CTK or enhancing the activity of CTK signaling pathway will promote the cell division and differentiation of floral meristem and increase the number of reproductive organs [45], also has study indicated that there was a positive correlation between grain number per panicle and CK content in rice [11]. Isopentenyltransferase (IPT) and cytokinin oxidase (CKX) are the main enzymes that maintain the homeostasis of endogenous cytokinin. Several studies have shown a correlation between CKX and yield. Using LOG as the promoter, *OsCKX4* antisense inhibition could increase the panicle size and spikelets number of plants without affecting other agronomic traits [46]. *OsCKX11* plays an important role in delaying leaf senescence, increasing seed number, and coordinating source and sink, *osckx11* showed significantly increased branching, tillering, and seed number compared to WT [47]. In this study, the endogenous cytokinin levels were assayed and found that the content of IP, IPA and Zeatin were all decreased, while DHZ was almost unchanged in the mutant (Fig. 6B-E). The expression level of several other members of the *OsCKX* family were also checked, the *OsCKX3*, *4*, 8 and *9* were downregulated, which were considered to be the reason why the DHZ content was unchanged in the mutant (Fig. S6). The DST-OsCKX2 model was previously reported to increase meristem activity, enhanced panicle branching, and a consequent increase of grain number [14]. The increased branches number of *abp1* may be due to the existence of other compensation pathways or other regulatory pathways (Fig. 1D, G). *FZP* is a critical gene in regulating panicle development, and the regulatory network is also extremely complex, in our RNA-seq data, the expression level of *TDD1* is also changed, which is related to the pathway of auxin synthesis (Fig. 4A), the formation and differentiation of meristem are regulated by the interaction of auxin and cytokinin [48, 49]. The promoter of *TDD1* also contains the motif of GCC-box, which can be specifically bound by FZP, whether there is a specific mechanism remains to be further studied. From the results and discussion above, a possible working model of how FZP might regulate the grain number was proposed, where FZP directly binds to the motif AGCCGCC in the *DST* promoter and represses its expression. Subsequently, *OsCKX2* expression level in inflorescence meristem of *fzp* mutant is up-regulated, and cytokinin level is decreased, resulting in decreased SAM activity, abnormal spikelet development, and down-regulated grain number per panicle. Additionally, we propose that *OsMADS-box* gene may also play a role (Fig. 6F).

## Conclusions

Collectively, our findings indicate that FZP represses the transcription of *DST* and regulates spikelet development by maintaining cytokinin homeostasis in inflorescence meristem via DST-CKX2 module. Our results indicate that FZP plays a critical role in regulating grain number through mediating cytokinin metabolism.

## Materials and methods

### Materials and growth

The *fzp* mutant was identified from EMS-treated seeds of the elite *indica* rice (*Oryza sativa*) variety YK17 (Zhongjiazao 17), the mutant stably inherited for multiple generations was applied for further experiments. The F_2_ mapping populations was derived from a cross between *fzp* mutant and tropical *japonica* rice variety D50. All plants were grown under natural conditions in the experimental fields of Zhejiang Academy of Agricultural Science in Hangzhou, China.

### Scanning electronic microscopy

For scanning electron microscopy analysis, the tissue samples collected from YK17 and *fzp* were fixed in 2.5% glutaraldehyde solution at 4 °C overnight, and the samples were rinsed three times with 0.1 M, pH7.0 phosphate buffer for 15 min each time. Then the samples were fixed in 1% osmic acid solution for 2 h, rinsed the samples three times with 0.1 M, pH7.0 phosphate buffer for three times. The samples were dehydrated with ethanol solutions of gradient concentrations, and then treated with 100% ethanol twice, 20 min each time. Drying process was performed in a Hitachi HCP-2 (Tokyo, Japan) critical point dryer. The samples were subsequently sputter-coated with gold and analyzed using the Hitachi SU-8010 scanning electron microscope (Tokyo, Japan).

### Map-based cloning and plant transformation

To determine the mutated gene, both parents and 20 individuals with the abnormal panicle phenotype were selected for initial linkage analysis. More than 200 simple sequences repeated (SSR) markers evenly distributed on 12 chromosomes of rice were used for preliminary mapping. The mutant control gene was initially located on chromosome 7. Using another 192 F_2_ plants, we delimited the gene to 995 kb region between MM0148 and MM3834. For the complementation of *fzp*, the *FZP* genomic DNA fragment containing 2.6-kb upstream sequences and 1.2-kb downstream sequence was inserted into the pCAMBIA2300 vector using kpnI and xbaI sites. The resulting plasmid was introduced into *fzp* mutant to generate transgenic plants [50]. All primers sequence were used in Supplemental Table 1.

### RNA extraction and qRT-PCR Analysis

Total RNA was extracted from different plant tissues except developing seeds of wild-type and *fzp* using Trizol reagent (Life Technologies). The method of RNA extraction from developing seeds was according to a modified SDS-Trizol method [51]. First Stand cDNA Synthesis Kit (TOYOBO) was used for RNA reverse transcription. The SYBR Green Real-time PCR Master Mix (Toyobo) was used for quantitative real-time PCR. The PCR programs were set according to a previous reported [52]. Experiments were performed with triplicates biological for each sample, the rice *UBQ* gene (LOC_Os03g13170) was used as internal control. The relative mRNA level of tested genes was normalized to UBQ and calculated by the 2^–DDCT^ method [53]. Primers for quantitative real-time PCR (qRT-PCR) analysis are listed in Supplementary Table S2.

### RNA-seq analysis

Young panicle samples (≤ 0.5 cm) of *fzp* and wild-type were collected, RNA extraction as previously described. Illumina Hiseq platform was used for sequencing in Novogene (China). Differentially expressed genes (DEGs) were identified as genes with absolute value of log2Fold changed ≥ 1 and q-value < 0.005 using DEGseq. For GO and KEGG analysis, Goseq and KOBAS (2.0) were used. The threshold of corrected *P*-value < 0.05 was used to identify significantly enriched GO terms and KEGG pathways.

### Subcellular localization of FZP

The full-length coding sequence of FZP without stop codon from YK17 was amplified by PCR. Then the PCR product was cloned into pAN580 vector to get the FZP-GFP construct [54]. The fusion plasmid and the marker plasmid D53-mCherry were co-transformed into rice protoplasts using polyethylene glycol according to a previous protocol [36, 55]. After 48 h incubating at 28^◦^C in the dark, the GFP signals were observed under a Zeiss LSM510 laser-scanning confocal microscopy (Karl Zeiss, Jena, Germany). The primers of this experiment were listed in Supplemental Table S2.

### Yeast one-hybrid assay

For yeast one-hybrid assay, the CDS of *FZP* was amplified and inserted into pB42AD, the *DST* promoter sequence was inserted into pLacZ2u to generate *ProDST: LacZ2u*. The plasmids were co-transformed into the yeast strain EGY48, the empty pB42AD in combination with the LacZ reporter constructs were used as negative controls [56]. The transformants were grew on SD/-Ura/-Trp medium for three days and then transferred to SD/-Ura/-Trp medium containing X-Gal for blue color test.

### Purification of tag-fused proteins and EMSA

Full length CDS of FZP was cloned into the Pmal-c2x to fuse with MBP tag, the recombinant protein was then transformed into *E. coli* DE3 cells and purified using MBPSep Dextrin 6FF Chromatography Column (YESEN Biotech, Shanghai, China) based on the manufacturer’s instructions. For EMSA assays, the probes of *DST* (62 nt in length) containing a motif AGCCGCC was commercially synthesized and labeled with an EMSA probe biotin labeling kit (Beyotime, Shanghai, China). Unlabeled DNA oligos were used as competitors. Reaction system was performed in a 10μL reaction volume which containing 2μL 5 × EMSA/Gel-shift binding buffer, 5 nmol purified recombinant protein and 2 nmol biotin-labeled probe. EMSA assays were performed with the chemiluminescent EMSA kit (Thermo, USA).

### Luciferase transient transcriptional activity assay

For dual-LUC assay, the 1.2 kb promoter sequence of DST was amplified and cloned into vector 190LUC as a reporter, and the full-length coding sequences of FZP was inserted into the control vector p*35S: NONE* as an effector. Total 10 μg of plasmid (effector, reporter and internal control) was used for the transformation in rice protoplast. Protoplast extraction and transformation were performed as previously described [57]. All the luciferase activities were measured using the Dual-Luciferase Reporter Assay System kit (Promega, Wisconsin, USA). Relative luciferase activity was calculated as the ratio of rLUS1 to rLUS2. Three biological replications were performed.

### Endogenous cytokinin analysis

For endogenous cytokinin level assay, 1 g young panicles (≤ 0.5 cm) were collected and pooled for measurements. Three biological replications were performed. Quantification of endogenous cytokinin was performed as previously described [15].

### Supplementary Information

**Additional file 1. **

**Additional file 2. **

**Additional file 3. **

**Additional file 4. **

## Data Availability

The original contributions presented in the study are included in the article and Supplementary materials, further inquiries can be directed to the corresponding author. Accession numbers Sequence data from this article for the cDNA and genomic DNA of FZP, OsCKX22 and DST can be found in the GenBank/EMBL/Gramene data libraries under accession numbers LOC_Os07g47330, LOC_Os01g10110 and LOC_Os03g57240, respectively.
