# Supplementary material for: FRIZZLE PANICLE (FZP) regulates rice spikelets development through modulating cytokinin metabolism
Source: BMC Plant Biol. 2023 Dec 16;23:650. doi: 10.1186/s12870-023-04671-4 (PMC10724965; doi:10.1186/s12870-023-04671-4)
Supplement: Supplementary file 1 — Additional file 1. [file 12870_2023_4671_MOESM1_ESM.pdf]

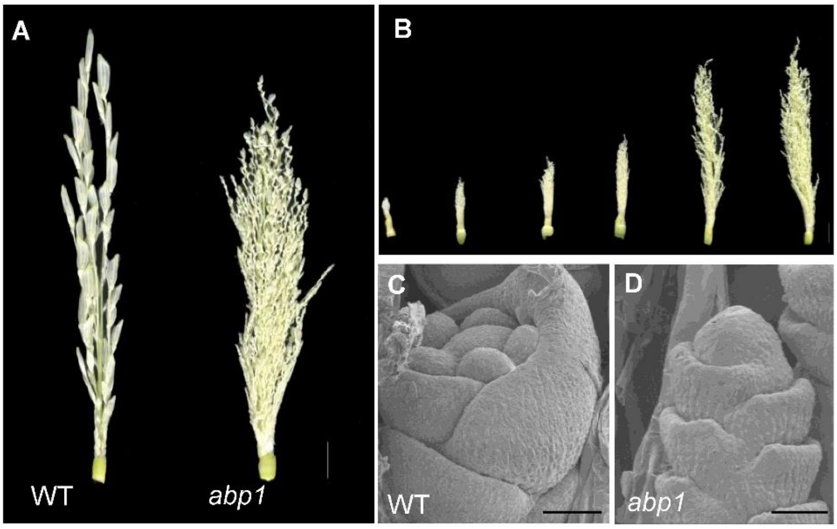

Fig. S1 Phenotypes observation of wild type and *abp1*.

A, B. Panicle phenotypes at different developmental stages. **C, D.** Scanning electronic microscope analysis of wild type and *abp1* panicle at early differentiation stage. Bar=500 μm.

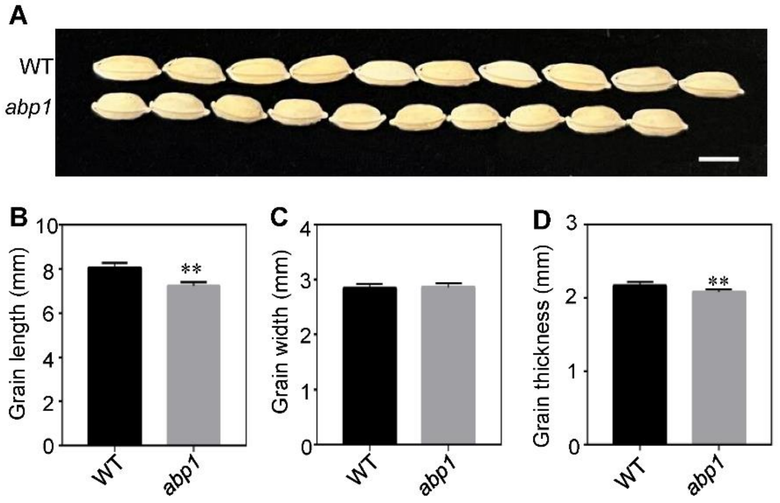

Fig. S2 Grain size analysis of wild type and *abp1*.

A. Grain size comparison of wild type (above) and *abp1* (below). Bar= 1cm. **B, C**. Comparisons of grain length, grain width and grain thickness between wild type and *abp1*. Data are shown as means ± SD (n = 10). The asterisk indicates the difference between wild type and *abp1* determined by Student's t-test (*P<0.05; **P<0.01).

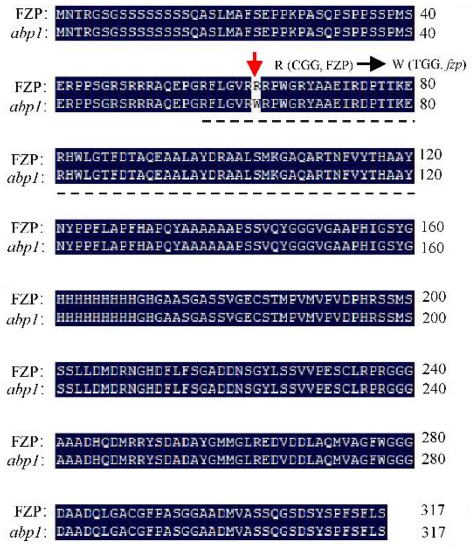

Fig. S3 Protein sequence alignment between FZP and *fzp*.

The dashed line represents the ERF domain.

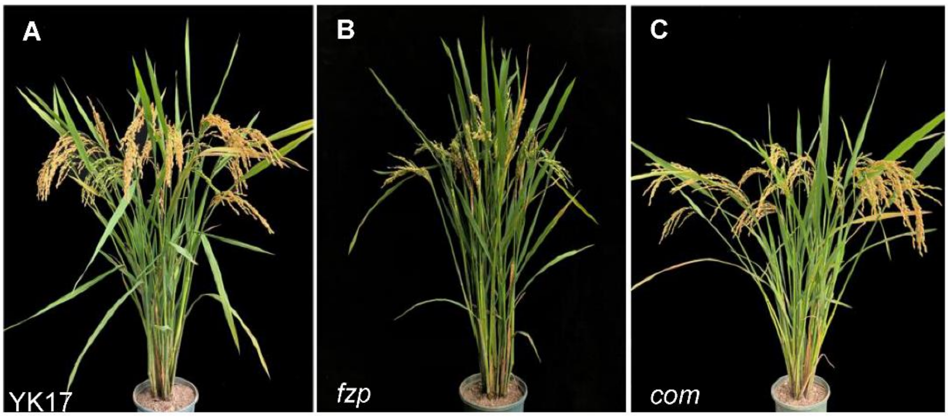

Fig. S4 Morphology of YK17, *fzp* and the genetic complementation lines (*com*).

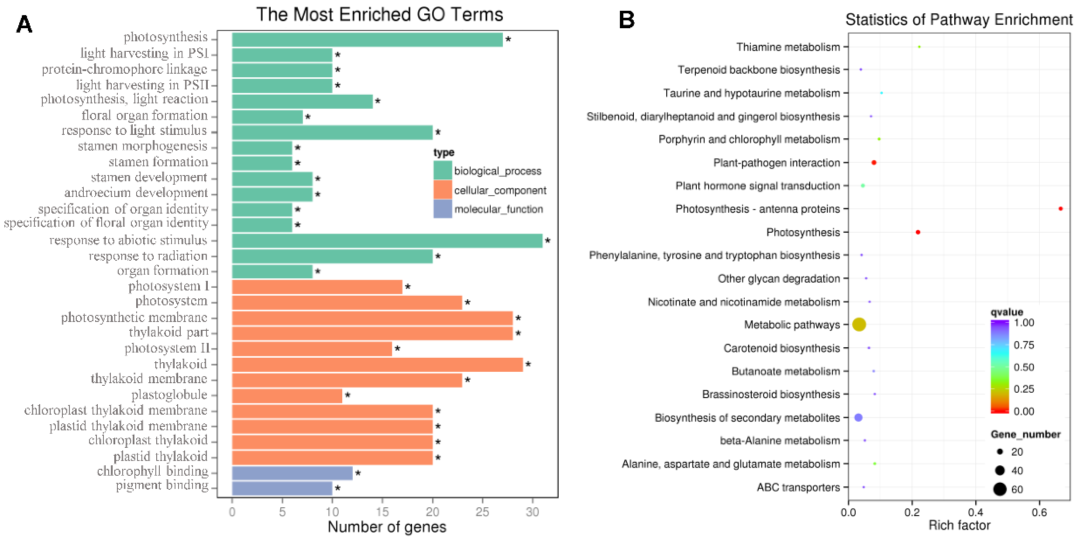

Fig. S5 GO and KEGG enrichment analysis of differentially expressed genes (DEGs) between WT and *fzp* (P≤0.05).

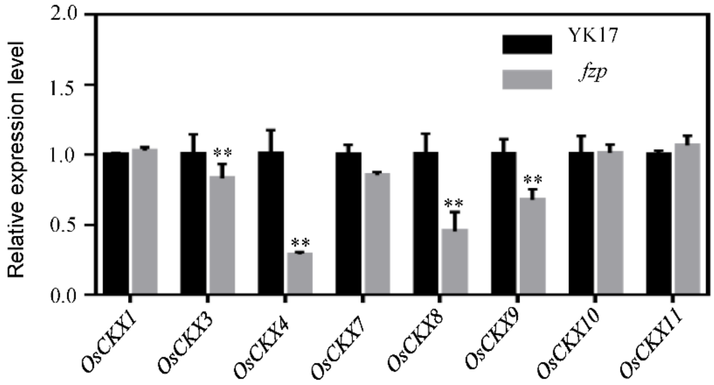

Fig. S6 Transcript levels of *OsCKXs* in the young panicles of WT and *fzp* revealed by qRT-PCR.

Data are means ± SD from three biological replicates. ** means significant difference between wild type and abp1 mutant as determined by the Student's *t*-test (P < 0.01).
