# Supplementary figures and images for: FRIZZLE PANICLE (FZP) regulates rice spikelets development through modulating cytokinin metabolism
Source: BMC Plant Biol. 2023 Dec 16;23:650. doi: 10.1186/s12870-023-04671-4 (PMC10724965; doi:10.1186/s12870-023-04671-4)

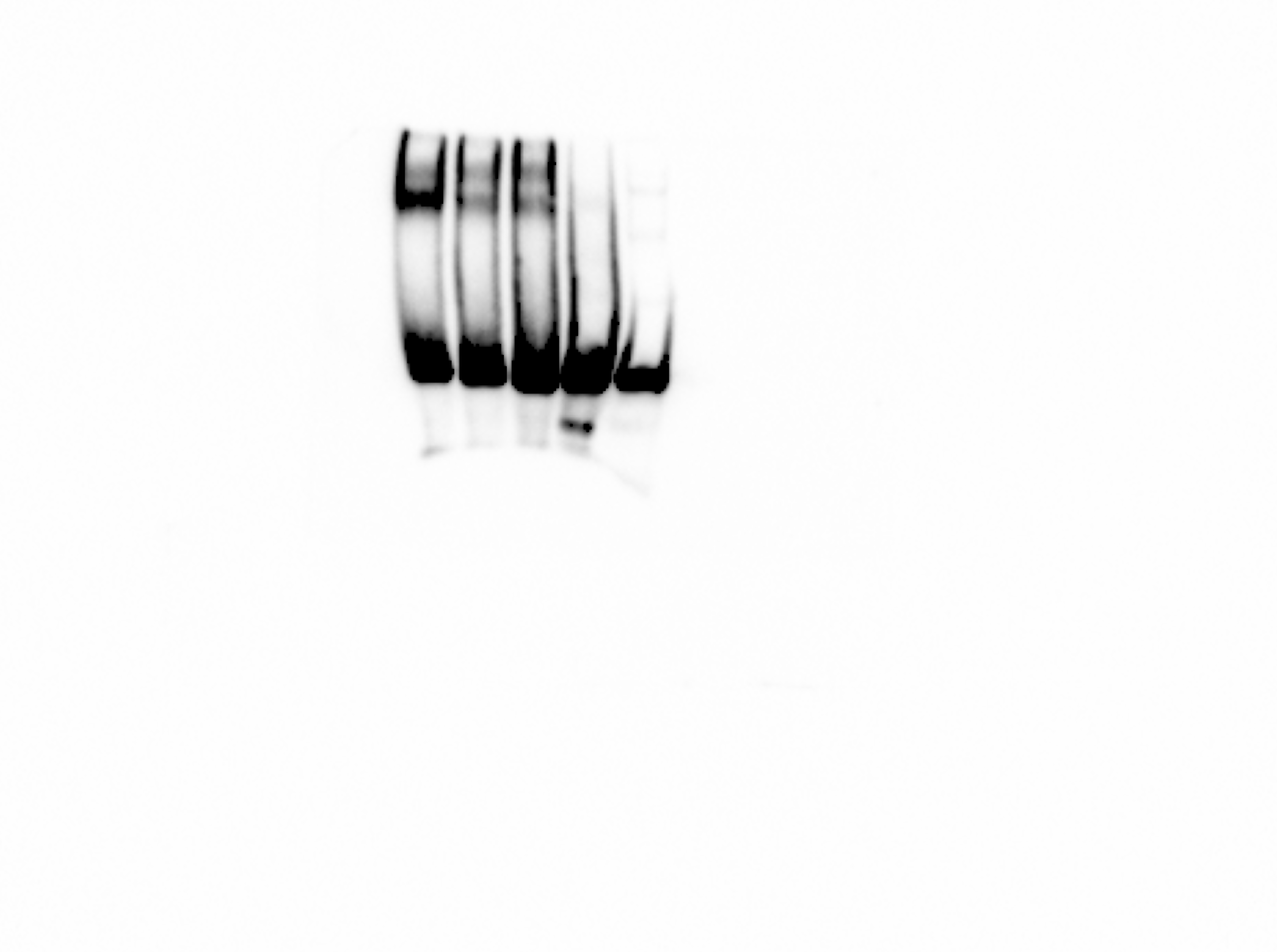

Supplement: Supplementary file 4 — Additional file 4. [file 12870_2023_4671_MOESM4_ESM.tif]
