# Supplementary material for: FRIZZLE PANICLE (FZP) regulates rice spikelets development through modulating cytokinin metabolism
Source: BMC Plant Biol. 2023 Dec 16;23:650. doi: 10.1186/s12870-023-04671-4 (PMC10724965; doi:10.1186/s12870-023-04671-4)
Supplement: Supplementary file 3 — Additional file 3. [file 12870_2023_4671_MOESM3_ESM.pdf]

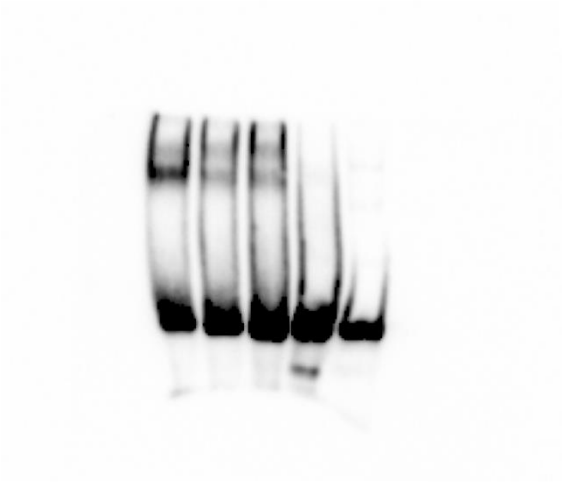

Image 1

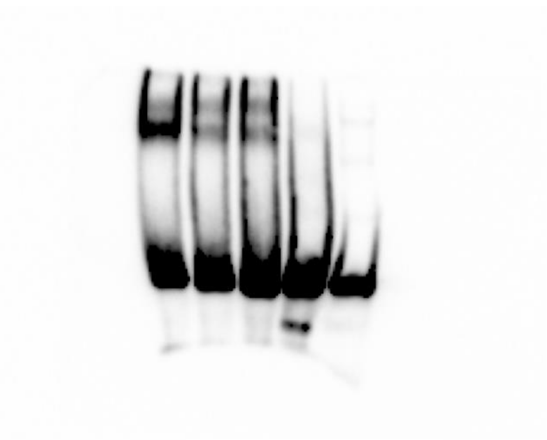

Image 2

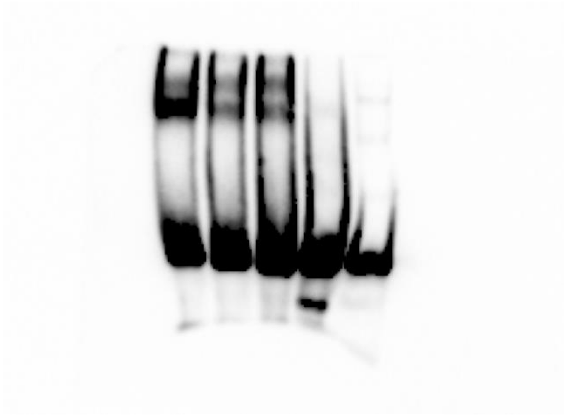

Image 3

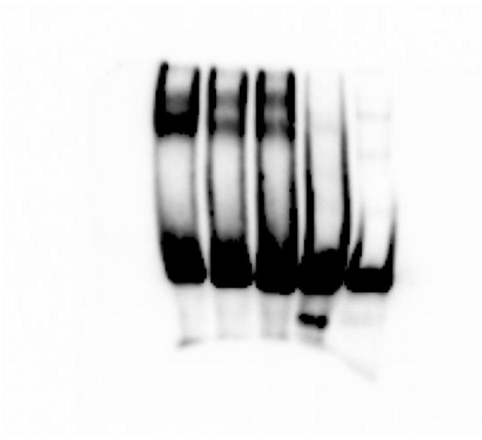

Image 4

Dear editor:

Image 1-4 showed the exposure photos of different periods within 1-10 seconds. The second picture was used in the manuscript, and the first picture was the photo taken at 1-second exposure time. The model number of gel imager used in the experiment was BIO-RAD universal hood III.

The cut angle was used to identify the direction of the gel during westernblot transmembrane.

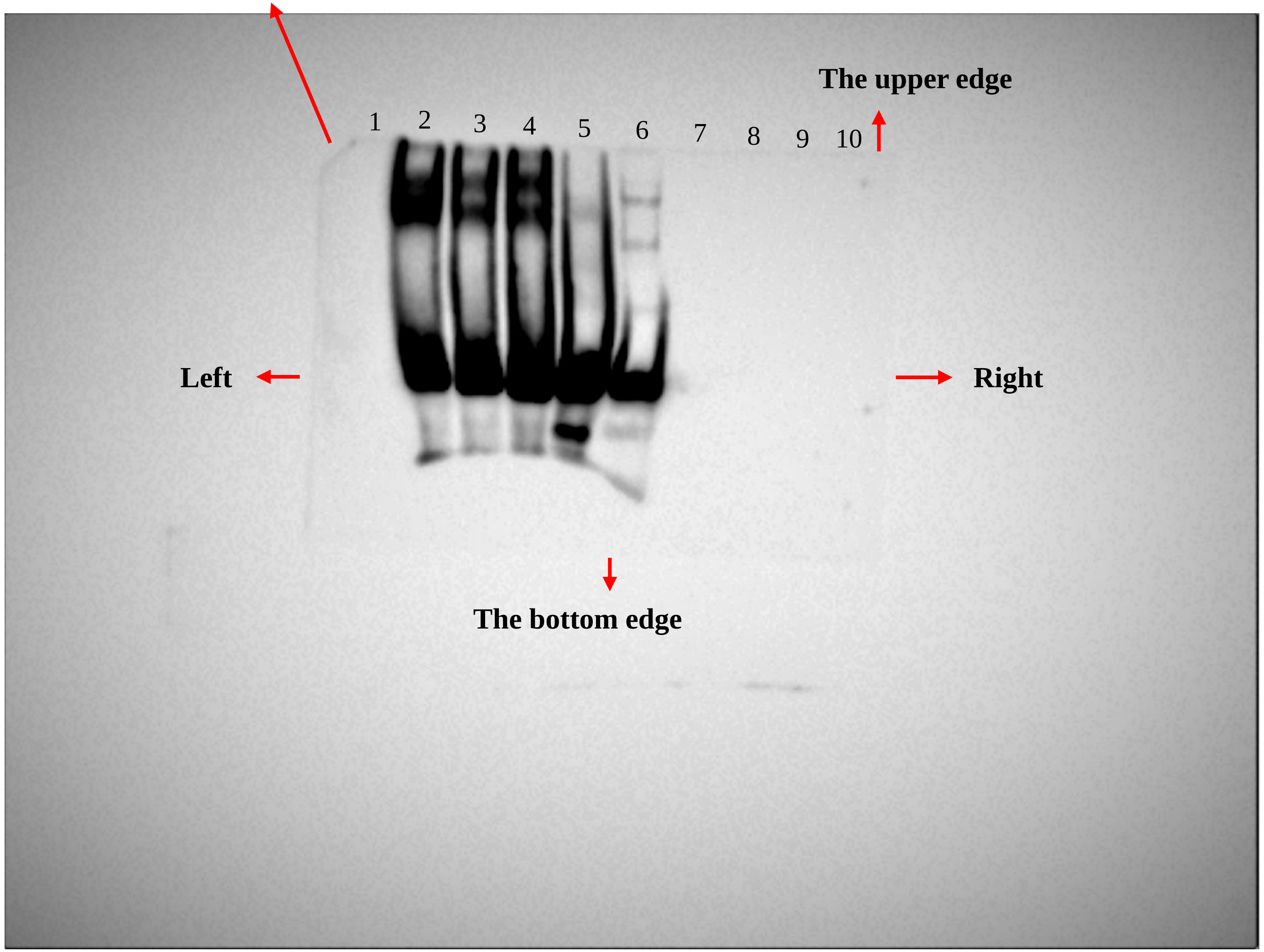

This image was used in the manuscript, a low-exposure, unadjusted contrast of the image on the left.

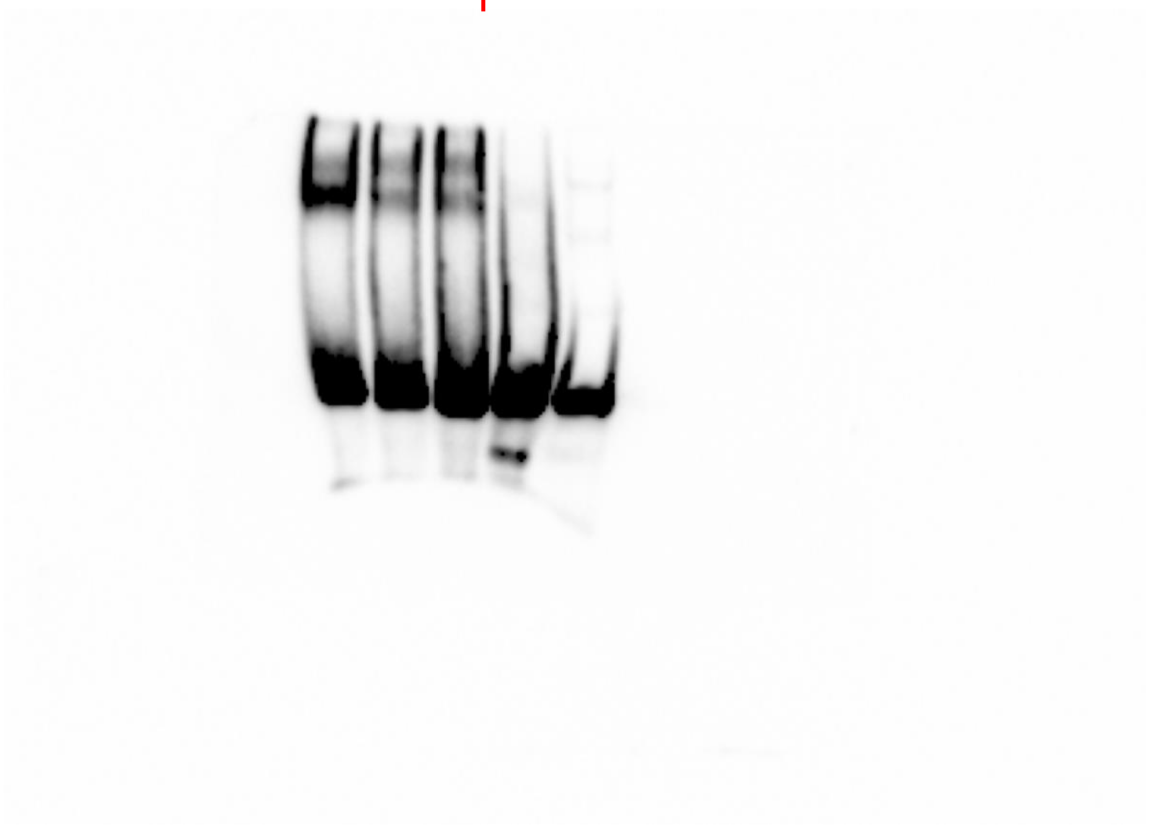

Dear editor:

By adjusting the contrast of the image with the longest exposure (the edges of the membrane can be faintly seen), the full length membrane can be clearly seen. The gel used in the experiment was 10-well gel. The first lane sample was diluted blue 1×EMSA/Gel-shift buffer, which was used to observe the progress of electrophoresis. Lane 7-10 was not sampled.
